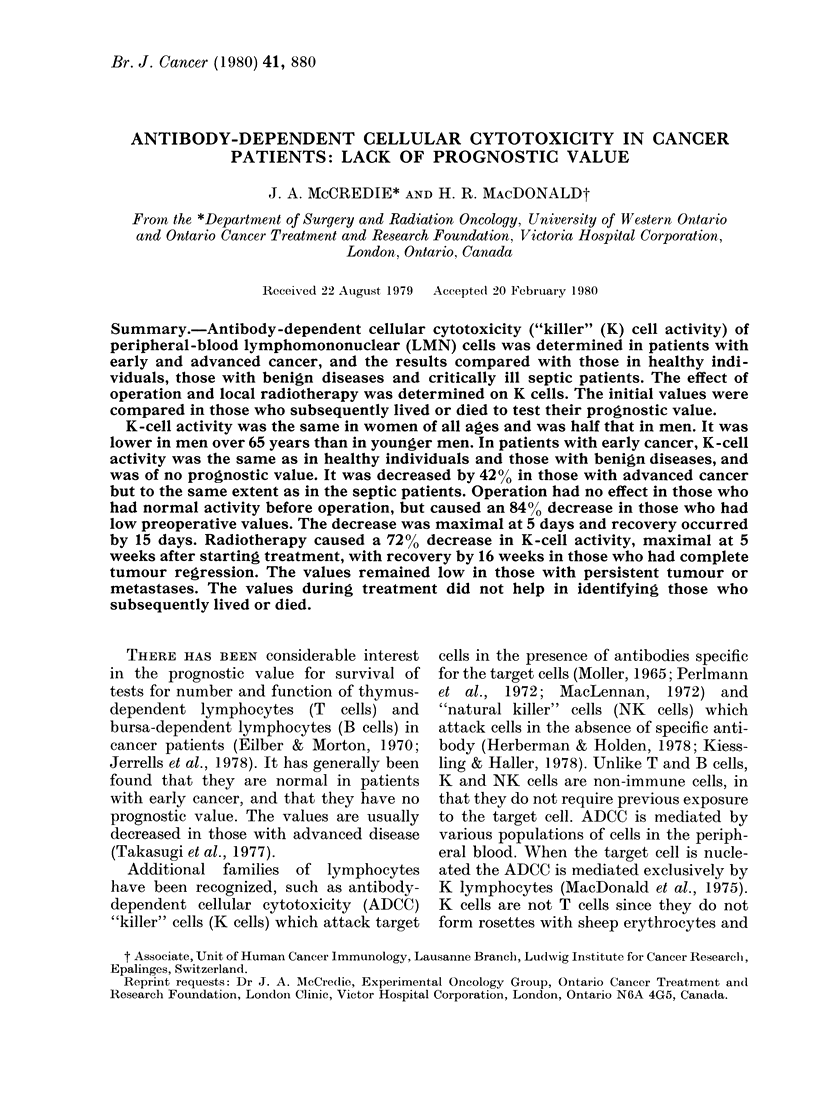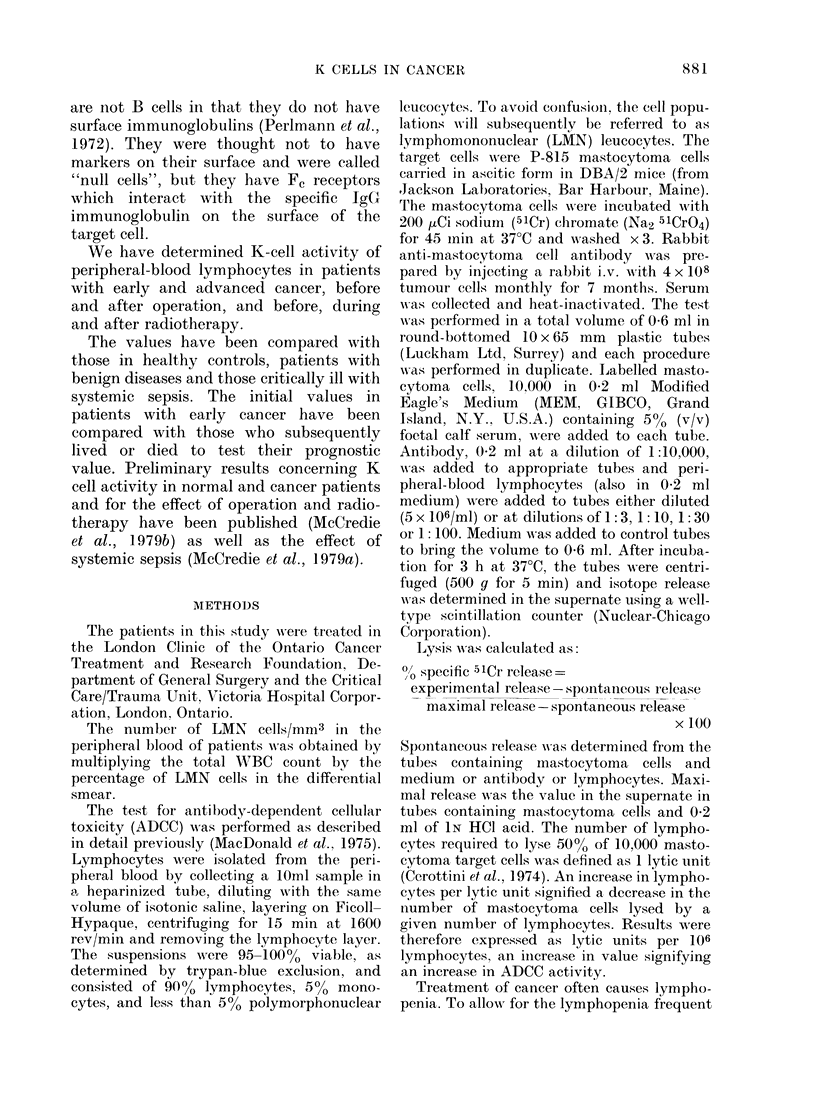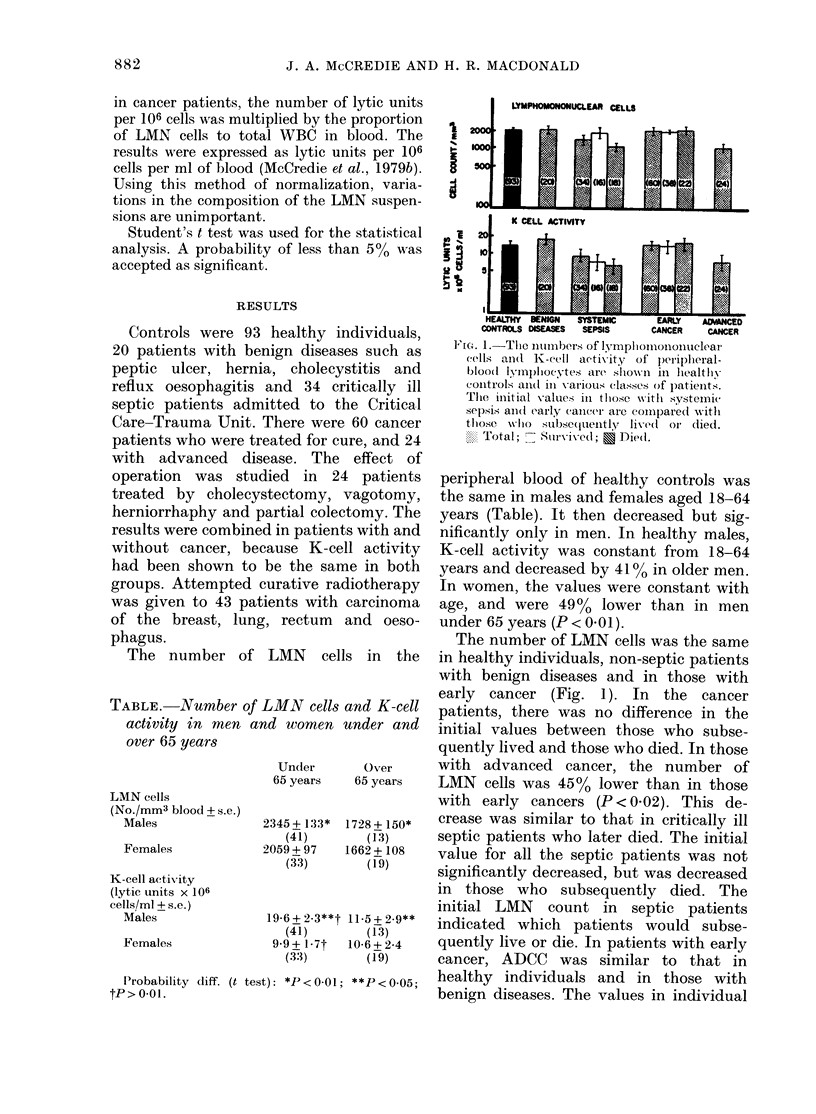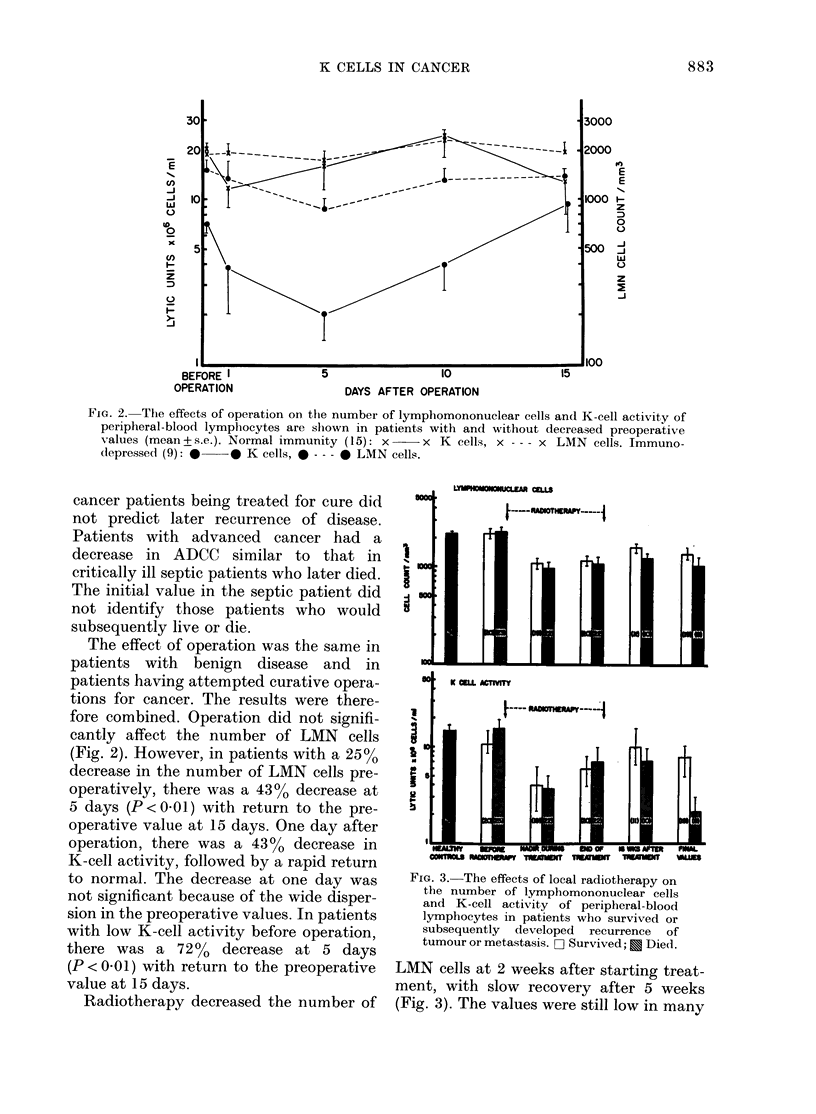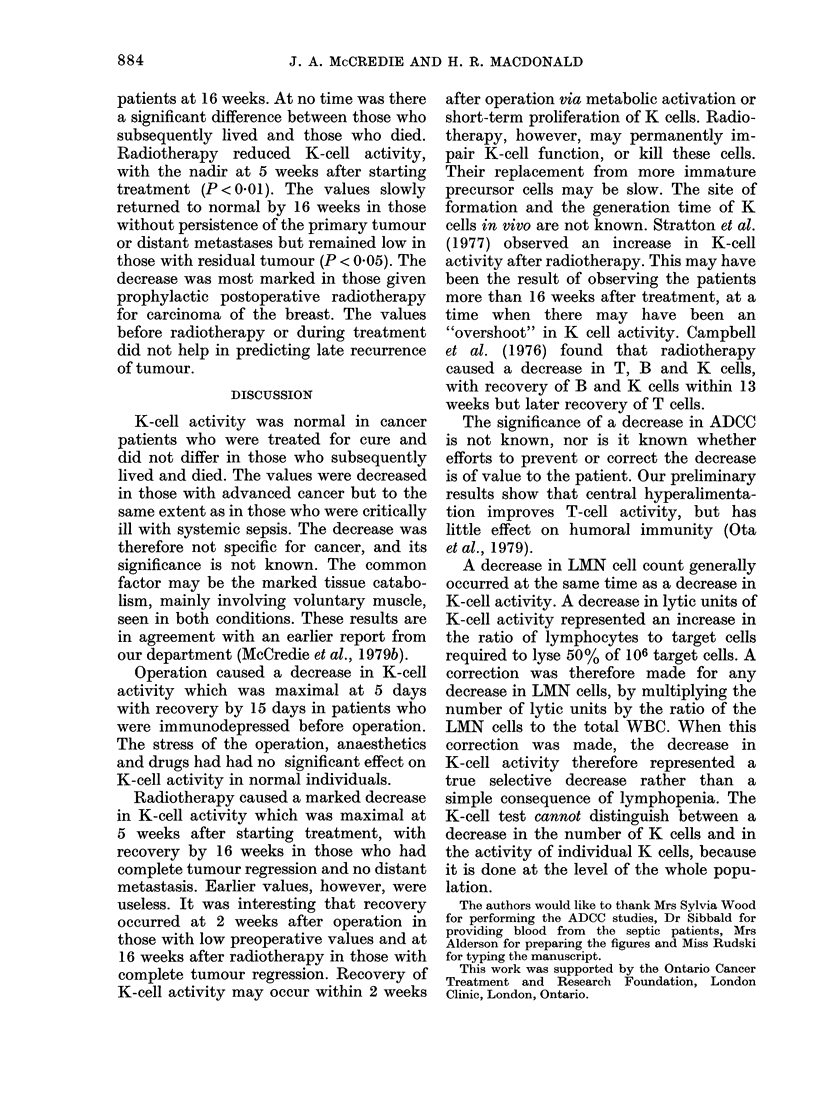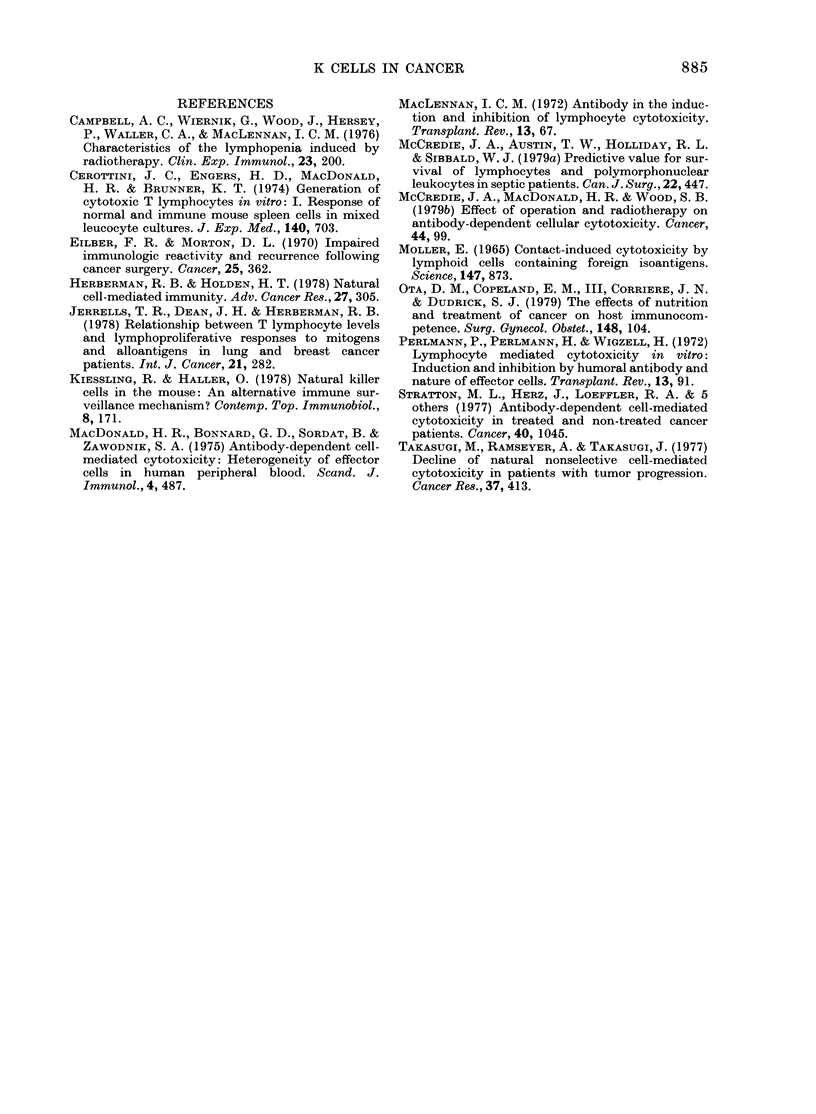# Antibody-dependent cellular cytotoxicity in cancer patients: lack of prognostic value

**DOI:** 10.1038/bjc.1980.164

**Published:** 1980-06

**Authors:** J. A. McCredie, H. R. MacDonald

## Abstract

Antibody-dependent cellular cytotoxicity (“killer” (K) cell activity) of peripheral-blood lymphomononuclear (LMN) cells was determined in patients with early and advanced cancer, and the results compared with those in healthy individuals, those with benign diseases and critically ill septic patients. The effect of operation and local radiotherapy was determined on K cells. The initial values were compared in those who subsequently lived or died to test their prognostic value.

K-cell activity was the same in women of all ages and was half that in men. It was lower in men over 65 years than in younger men. In patients with early cancer, K-cell activity was the same as in healthy individuals and those with benign diseases, and was of no prognostic value. It was decreased by 42% in those with advanced cancer but to the same extent as in the septic patients. Operation had no effect in those who had normal activity before operation, but caused an 84% decrease in those who had low preoperative values. The decrease was maximal at 5 days and recovery occurred by 15 days. Radiotherapy caused a 72% decrease in K-cell activity, maximal at 5 weeks after starting treatment, with recovery by 16 weeks in those who had complete tumour regression. The values remained low in those with persistent tumour or metastases. The values during treatment did not help in identifying those who subsequently lived or died.


*Br. J. Cancer* (1980) **41**, 880

### ANTIBODY-DEPENDENT CELLULAR CYTOTOXICITY IN CANCER PATIENTS: LACK OF PROGNOSTIC VALUE

J. A. McCREDIE* AND H. R. MACDONALD†

*From the *Department of Surgery and Radiation Oncology, University of Western Ontario and Ontario Cancer Treatment and Research Foundation, Victoria Hospital Corporation, London, Ontario, Canada*

Received 22 August 1979 Accepted 20 February 1980

**Summary.—Antibody-dependent cellular cytotoxicity ("killer" (K) cell activity) of peripheral-blood lymphomononuclear (LMN) cells was determined in patients with early and advanced cancer, and the results compared with those in healthy individuals, those with benign diseases and critically ill septic patients. The effect of operation and local radiotherapy was determined on K cells. The initial values were compared in those who subsequently lived or died to test their prognostic value.**

**K-cell activity was the same in women of all ages and was half that in men. It was lower in men over 65 years than in younger men. In patients with early cancer, K-cell activity was the same as in healthy individuals and those with benign diseases, and was of no prognostic value. It was decreased by 42% in those with advanced cancer but to the same extent as in the septic patients. Operation had no effect in those who had normal activity before operation, but caused an 84% decrease in those who had low preoperative values. The decrease was maximal at 5 days and recovery occurred by 15 days. Radiotherapy caused a 72% decrease in K-cell activity, maximal at 5 weeks after starting treatment, with recovery by 16 weeks in those who had complete tumour regression. The values remained low in those with persistent tumour or metastases. The values during treatment did not help in identifying those who subsequently lived or died.**

THERE HAS BEEN considerable interest in the prognostic value for survival of tests for number and function of thymus-dependent lymphocytes (T cells) and bursa-dependent lymphocytes (B cells) in cancer patients (Eilber & Morton, 1970; Jerrells *et al.*, 1978). It has generally been found that they are normal in patients with early cancer, and that they have no prognostic value. The values are usually decreased in those with advanced disease (Takasugi *et al.*, 1977).

Additional families of lymphocytes have been recognized, such as antibody-dependent cellular cytotoxicity (ADCC) "killer" cells (K cells) which attack target cells in the presence of antibodies specific for the target cells (Moller, 1965; Perlmann *et al.*, 1972; MacLennan, 1972) and "natural killer" cells (NK cells) which attack cells in the absence of specific antibody (Herberman & Holden, 1978; Kiessling & Haller, 1978). Unlike T and B cells, K and NK cells are non-immune cells, in that they do not require previous exposure to the target cell. ADCC is mediated by various populations of cells in the peripheral blood. When the target cell is nucleated the ADCC is mediated exclusively by K lymphocytes (MacDonald *et al.*, 1975). K cells are not T cells since they do not form rosettes with sheep erythrocytes and

† Associate, Unit of Human Cancer Immunology, Lausanne Branch, Ludwig Institute for Cancer Research, Epalinges, Switzerland.

Reprint requests: Dr J. A. McCredie, Experimental Oncology Group, Ontario Cancer Treatment and Research Foundation, London Clinic, Victor Hospital Corporation, London, Ontario N6A 4G5, Canada.

are not B cells in that they do not have surface immunoglobulins (Perlmann *et al.*, 1972). They were thought not to have markers on their surface and were called "null cells", but they have $F_c$ receptors which interact with the specific IgG immunoglobulin on the surface of the target cell.

We have determined K-cell activity of peripheral-blood lymphocytes in patients with early and advanced cancer, before and after operation, and before, during and after radiotherapy.

The values have been compared with those in healthy controls, patients with benign diseases and those critically ill with systemic sepsis. The initial values in patients with early cancer have been compared with those who subsequently lived or died to test their prognostic value. Preliminary results concerning K cell activity in normal and cancer patients and for the effect of operation and radiotherapy have been published (McCredie *et al.*, 1979*b*) as well as the effect of systemic sepsis (McCredie *et al.*, 1979*a*).

### METHODS

The patients in this study were treated in the London Clinic of the Ontario Cancer Treatment and Research Foundation, Department of General Surgery and the Critical Care/Trauma Unit, Victoria Hospital Corporation, London, Ontario.

The number of LMN cells/mm³ in the peripheral blood of patients was obtained by multiplying the total WBC count by the percentage of LMN cells in the differential smear.

The test for antibody-dependent cellular toxicity (ADCC) was performed as described in detail previously (MacDonald *et al.*, 1975). Lymphocytes were isolated from the peripheral blood by collecting a 10ml sample in a heparinized tube, diluting with the same volume of isotonic saline, layering on Ficoll–Hypaque, centrifuging for 15 min at 1600 rev/min and removing the lymphocyte layer. The suspensions were 95–100% viable, as determined by trypan-blue exclusion, and consisted of 90% lymphocytes, 5% monocytes, and less than 5% polymorphonuclear leucocytes. To avoid confusion, the cell populations will subsequently be referred to as lymphomononuclear (LMN) leucocytes. The target cells were P-815 mastocytoma cells carried in ascitic form in DBA/2 mice (from Jackson Laboratories, Bar Harbour, Maine). The mastocytoma cells were incubated with 200 μCi sodium ($^{51}$Cr) chromate ($Na_2{}^{51}CrO_4$) for 45 min at 37°C and washed ×3. Rabbit anti-mastocytoma cell antibody was prepared by injecting a rabbit i.v. with $4 \times 10^8$ tumour cells monthly for 7 months. Serum was collected and heat-inactivated. The test was performed in a total volume of 0·6 ml in round-bottomed 10×65 mm plastic tubes (Luckham Ltd, Surrey) and each procedure was performed in duplicate. Labelled mastocytoma cells, 10,000 in 0·2 ml Modified Eagle's Medium (MEM, GIBCO, Grand Island, N.Y., U.S.A.) containing 5% (v/v) foetal calf serum, were added to each tube. Antibody, 0·2 ml at a dilution of 1:10,000, was added to appropriate tubes and peripheral-blood lymphocytes (also in 0·2 ml medium) were added to tubes either diluted ($5 \times 10^6$/ml) or at dilutions of 1:3, 1:10, 1:30 or 1:100. Medium was added to control tubes to bring the volume to 0·6 ml. After incubation for 3 h at 37°C, the tubes were centrifuged (500 *g* for 5 min) and isotope release was determined in the supernate using a well-type scintillation counter (Nuclear-Chicago Corporation).

Lysis was calculated as:

$$\% \text{ specific } {}^{51}\text{Cr release} = \frac{\text{experimental release} - \text{spontaneous release}}{\text{maximal release} - \text{spontaneous release}} \times 100$$

Spontaneous release was determined from the tubes containing mastocytoma cells and medium or antibody or lymphocytes. Maximal release was the value in the supernate in tubes containing mastocytoma cells and 0·2 ml of 1N HCl acid. The number of lymphocytes required to lyse 50% of 10,000 mastocytoma target cells was defined as 1 lytic unit (Cerottini *et al.*, 1974). An increase in lymphocytes per lytic unit signified a decrease in the number of mastocytoma cells lysed by a given number of lymphocytes. Results were therefore expressed as lytic units per $10^6$ lymphocytes, an increase in value signifying an increase in ADCC activity.

Treatment of cancer often causes lymphopenia. To allow for the lymphopenia frequent in cancer patients, the number of lytic units per $10^6$ cells was multiplied by the proportion of LMN cells to total WBC in blood. The results were expressed as lytic units per $10^6$ cells per ml of blood (McCredie *et al.*, 1979*b*). Using this method of normalization, variations in the composition of the LMN suspensions are unimportant.

Student's *t* test was used for the statistical analysis. A probability of less than 5% was accepted as significant.

### RESULTS

Controls were 93 healthy individuals, 20 patients with benign diseases such as peptic ulcer, hernia, cholecystitis and reflux oesophagitis and 34 critically ill septic patients admitted to the Critical Care–Trauma Unit. There were 60 cancer patients who were treated for cure, and 24 with advanced disease. The effect of operation was studied in 24 patients treated by cholecystectomy, vagotomy, herniorrhaphy and partial colectomy. The results were combined in patients with and without cancer, because K-cell activity had been shown to be the same in both groups. Attempted curative radiotherapy was given to 43 patients with carcinoma of the breast, lung, rectum and oesophagus.

The number of LMN cells in the peripheral blood of healthy controls was the same in males and females aged 18–64 years (Table). It then decreased but significantly only in men. In healthy males, K-cell activity was constant from 18–64 years and decreased by 41% in older men. In women, the values were constant with age, and were 49% lower than in men under 65 years ($P < 0{\cdot}01$).

TABLE.—*Number of LMN cells and K-cell activity in men and women under and over 65 years*

| | Under 65 years | Over 65 years |
|---|---|---|
| LMN cells (No./mm³ blood ± s.e.) | | |
| Males | 2345 ± 133* (41) | 1728 ± 150* (13) |
| Females | 2059 ± 97 (33) | 1662 ± 108 (19) |
| K-cell activity (lytic units × $10^6$ cells/ml ± s.e.) | | |
| Males | 19·6 ± 2·3**† (41) | 11·5 ± 2·9** (13) |
| Females | 9·9 ± 1·7† (33) | 10·6 ± 2·4 (19) |

Probability diff. (*t* test): *$P < 0{\cdot}01$; **$P < 0{\cdot}05$; †$P > 0{\cdot}01$.

FIG. 1.—The numbers of lymphomononuclear cells and K-cell activity of peripheral-blood lymphocytes are shown in healthy controls and in various classes of patients. The initial values in those with systemic sepsis and early cancer are compared with those who subsequently lived or died. ░ Total; □ Survived; ▧ Died.

The number of LMN cells was the same in healthy individuals, non-septic patients with benign diseases and in those with early cancer (Fig. 1). In the cancer patients, there was no difference in the initial values between those who subsequently lived and those who died. In those with advanced cancer, the number of LMN cells was 45% lower than in those with early cancers ($P < 0{\cdot}02$). This decrease was similar to that in critically ill septic patients who later died. The initial value for all the septic patients was not significantly decreased, but was decreased in those who subsequently died. The initial LMN count in septic patients indicated which patients would subsequently live or die. In patients with early cancer, ADCC was similar to that in healthy individuals and in those with benign diseases. The values in individual

FIG. 2.—The effects of operation on the number of lymphomononuclear cells and K-cell activity of peripheral-blood lymphocytes are shown in patients with and without decreased preoperative values (mean ± s.e.). Normal immunity (15): ×——× K cells, × - - - × LMN cells. Immunodepressed (9): ●——● K cells, ● - - - ● LMN cells.

cancer patients being treated for cure did not predict later recurrence of disease. Patients with advanced cancer had a decrease in ADCC similar to that in critically ill septic patients who later died. The initial value in the septic patient did not identify those patients who would subsequently live or die.

The effect of operation was the same in patients with benign disease and in patients having attempted curative operations for cancer. The results were therefore combined. Operation did not significantly affect the number of LMN cells (Fig. 2). However, in patients with a 25% decrease in the number of LMN cells preoperatively, there was a 43% decrease at 5 days ($P < 0{\cdot}01$) with return to the preoperative value at 15 days. One day after operation, there was a 43% decrease in K-cell activity, followed by a rapid return to normal. The decrease at one day was not significant because of the wide dispersion in the preoperative values. In patients with low K-cell activity before operation, there was a 72% decrease at 5 days ($P < 0{\cdot}01$) with return to the preoperative value at 15 days.

Radiotherapy decreased the number of

FIG. 3.—The effects of local radiotherapy on the number of lymphomononuclear cells and K-cell activity of peripheral-blood lymphocytes in patients who survived or subsequently developed recurrence of tumour or metastasis. □ Survived; ▨ Died.

LMN cells at 2 weeks after starting treatment, with slow recovery after 5 weeks (Fig. 3). The values were still low in many patients at 16 weeks. At no time was there a significant difference between those who subsequently lived and those who died. Radiotherapy reduced K-cell activity, with the nadir at 5 weeks after starting treatment ($P < 0{\cdot}01$). The values slowly returned to normal by 16 weeks in those without persistence of the primary tumour or distant metastases but remained low in those with residual tumour ($P < 0{\cdot}05$). The decrease was most marked in those given prophylactic postoperative radiotherapy for carcinoma of the breast. The values before radiotherapy or during treatment did not help in predicting late recurrence of tumour.

### DISCUSSION

K-cell activity was normal in cancer patients who were treated for cure and did not differ in those who subsequently lived and died. The values were decreased in those with advanced cancer but to the same extent as in those who were critically ill with systemic sepsis. The decrease was therefore not specific for cancer, and its significance is not known. The common factor may be the marked tissue catabolism, mainly involving voluntary muscle, seen in both conditions. These results are in agreement with an earlier report from our department (McCredie *et al.*, 1979*b*).

Operation caused a decrease in K-cell activity which was maximal at 5 days with recovery by 15 days in patients who were immunodepressed before operation. The stress of the operation, anaesthetics and drugs had had no significant effect on K-cell activity in normal individuals.

Radiotherapy caused a marked decrease in K-cell activity which was maximal at 5 weeks after starting treatment, with recovery by 16 weeks in those who had complete tumour regression and no distant metastasis. Earlier values, however, were useless. It was interesting that recovery occurred at 2 weeks after operation in those with low preoperative values and at 16 weeks after radiotherapy in those with complete tumour regression. Recovery of K-cell activity may occur within 2 weeks after operation *via* metabolic activation or short-term proliferation of K cells. Radiotherapy, however, may permanently impair K-cell function, or kill these cells. Their replacement from more immature precursor cells may be slow. The site of formation and the generation time of K cells *in vivo* are not known. Stratton *et al.* (1977) observed an increase in K-cell activity after radiotherapy. This may have been the result of observing the patients more than 16 weeks after treatment, at a time when there may have been an "overshoot" in K cell activity. Campbell *et al.* (1976) found that radiotherapy caused a decrease in T, B and K cells, with recovery of B and K cells within 13 weeks but later recovery of T cells.

The significance of a decrease in ADCC is not known, nor is it known whether efforts to prevent or correct the decrease is of value to the patient. Our preliminary results show that central hyperalimentation improves T-cell activity, but has little effect on humoral immunity (Ota *et al.*, 1979).

A decrease in LMN cell count generally occurred at the same time as a decrease in K-cell activity. A decrease in lytic units of K-cell activity represented an increase in the ratio of lymphocytes to target cells required to lyse 50% of $10^6$ target cells. A correction was therefore made for any decrease in LMN cells, by multiplying the number of lytic units by the ratio of the LMN cells to the total WBC. When this correction was made, the decrease in K-cell activity therefore represented a true selective decrease rather than a simple consequence of lymphopenia. The K-cell test *cannot* distinguish between a decrease in the number of K cells and in the activity of individual K cells, because it is done at the level of the whole population.

The authors would like to thank Mrs Sylvia Wood for performing the ADCC studies, Dr Sibbald for providing blood from the septic patients, Mrs Alderson for preparing the figures and Miss Rudski for typing the manuscript.

This work was supported by the Ontario Cancer Treatment and Research Foundation, London Clinic, London, Ontario.

### REFERENCES

CAMPBELL, A. C., WIERNIK, G., WOOD, J., HERSEY, P., WALLER, C. A., & MACLENNAN, I. C. M. (1976) Characteristics of the lymphopenia induced by radiotherapy. *Clin. Exp. Immunol.*, **23**, 200.

CEROTTINI, J. C., ENGERS, H. D., MACDONALD, H. R. & BRUNNER, K. T. (1974) Generation of cytotoxic T lymphocytes *in vitro*: I. Response of normal and immune mouse spleen cells in mixed leucocyte cultures. *J. Exp. Med.*, **140**, 703.

EILBER, F. R. & MORTON, D. L. (1970) Impaired immunologic reactivity and recurrence following cancer surgery. *Cancer*, **25**, 362.

HERBERMAN, R. B. & HOLDEN, H. T. (1978) Natural cell-mediated immunity. *Adv. Cancer Res.*, **27**, 305.

JERRELLS, T. R., DEAN, J. H. & HERBERMAN, R. B. (1978) Relationship between T lymphocyte levels and lymphoproliferative responses to mitogens and alloantigens in lung and breast cancer patients. *Int. J. Cancer*, **21**, 282.

KIESSLING, R. & HALLER, O. (1978) Natural killer cells in the mouse: An alternative immune surveillance mechanism? *Contemp. Top. Immunobiol.*, **8**, 171.

MACDONALD, H. R., BONNARD, G. D., SORDAT, B. & ZAWODNIK, S. A. (1975) Antibody-dependent cell-mediated cytotoxicity: Heterogeneity of effector cells in human peripheral blood. *Scand. J. Immunol.*, **4**, 487.

MACLENNAN, I. C. M. (1972) Antibody in the induction and inhibition of lymphocyte cytotoxicity. *Transplant. Rev.*, **13**, 67.

MCCREDIE, J. A., AUSTIN, T. W., HOLLIDAY, R. L. & SIBBALD, W. J. (1979*a*) Predictive value for survival of lymphocytes and polymorphonuclear leukocytes in septic patients. *Can. J. Surg.*, **22**, 447.

MCCREDIE, J. A., MACDONALD, H. R. & WOOD, S. B. (1979*b*) Effect of operation and radiotherapy on antibody-dependent cellular cytotoxicity. *Cancer*, **44**, 99.

MOLLER, E. (1965) Contact-induced cytotoxicity by lymphoid cells containing foreign isoantigens. *Science*, **147**, 873.

OTA, D. M., COPELAND, E. M., III, CORRIERE, J. N. & DUDRICK, S. J. (1979) The effects of nutrition and treatment of cancer on host immunocompetence. *Surg. Gynecol. Obstet.*, **148**, 104.

PERLMANN, P., PERLMANN, H. & WIGZELL, H. (1972) Lymphocyte mediated cytotoxicity *in vitro*: Induction and inhibition by humoral antibody and nature of effector cells. *Transplant. Rev.*, **13**, 91.

STRATTON, M. L., HERZ, J., LOEFFLER, R. A. & 5 others (1977) Antibody-dependent cell-mediated cytotoxicity in treated and non-treated cancer patients. *Cancer*, **40**, 1045.

TAKASUGI, M., RAMSEYER, A. & TAKASUGI, J. (1977) Decline of natural nonselective cell-mediated cytotoxicity in patients with tumor progression. *Cancer Res.*, **37**, 413.